# Plasma elaidic acid level is associated with periodontal health in American adults: A cross-sectional study

**DOI:** 10.3389/fnut.2022.1034841

**Published:** 2022-12-08

**Authors:** Hu Jiaxin, Chen Zhu, Yang Jing, Shi Qianhui, Jin Fuqian, Pang Qiyuan, Luo Yi, Song Jukun

**Affiliations:** ^1^Guiyang Hospital of Stomatology, Guiyang, Guizhou, China; ^2^School of Stomatology, Zunyi Medical University, Zunyi, Guizhou, China; ^3^The Affiliated Stomatological Hospital and Stomatology of Guizhou Medical University, Guizhou Medical University, Guiyang, China

**Keywords:** NHANES, inflammation, periodontal health, trans fatty acids, elaidic acid, trans-9-octadecenoic acid, TFA

## Abstract

**Background:**

Whether there is an association between TFAs and periodontitis is unclear. The purpose of this study was to investigate the relationship between moderate/severe periodontitis and plasma level of elaidic acid, a major trans-fatty acid component, in American adults.

**Methods:**

The National Health and Nutrition Examination Survey (NHANES) years 2009–2010 were used to screen a total of 1,610 people. The independent variable of interest is plasma elaidic acid level, the dependent variable is periodontitis, and the covariates include socio-demographic variables, lifestyle variables, systemic diseases, etc. The distribution of variables in the covariate differences between the different independent groups according to tertile was investigated using a multiple linear regression model. To examine the association between plasma elaidic acid levels and moderate/severe periodontitis, three models were used.

**Results:**

Multiple logistic regression analysis showed a significant association between plasma elaidic acid level and moderate/severe periodontitis after adjustment for potential confounders (OR = 1.021, 95%CI: 1.004–1.039, *P* = 0.01394). Subjects with the highest tertile of plasma elaidic acid levels were 51.9% more likely to have periodontitis compared with subjects with the lowest tertile of plasma elaidic acid levels (OR = 1.519, 95% CI: 1.136–2.030, *P* = 0.00477). No possible sources of heterogeneity were identified in the subgroup analyses.

**Conclusion:**

Plasma elaidic acid levels are associated with periodontal disease in American adults.

## Introduction

Periodontitis is a chronic inflammation and a destructive disease, eventually leading to the gradual loosening of teeth if not diagnosed and treated (1). Massive tooth loss can result in poor diet, which can cause weight loss (2). It may also impair speech and appearance, which could harm one’s mental health and sense of self. Approximately 46% of American adults aged 30 and older have periodontitis, which is a high prevalence (3). It was ranked as the sixth most common illness worldwide.

The main pathogenesis of periodontitis is the accumulation of pathogenic microorganisms in plaque, which leads to host inflammatory immune response (4–6). Usually, the periodontitis is controlled by mechanical removal of tartar, and severe cases can be further treated by surgery. However, numerous risk factors, such as a person’s lifestyle, underlying illnesses, and sociological factors, can aggravate that degree of periodontitis or increase the probability of occurrence of periodontitis (7–10). And if the aforementioned risk factors are disregarded, the government may struggle to effectively address the national prevalence of periodontitis by raising awareness of periodontal treatment alone (11, 12).

An inflammatory dietary pattern is linked to the prevalence of periodontitis, according to recent studies (13–17). It should be noted that the majority of trans fatty acid (TFA)-rich foods, including cookies, candies, chocolate, potato chips, French fries, other fried foods, and fast food, are inflammatory foods (18, 19).

TFA are non-conjugated unsaturated fatty acids that contain one or more trans double bonds in the molecule. Only a minor quantity of TFA are formed in ruminant rumen fermentation (20), while the majority of TFA are produced in industrial processing by partial hydrogenation of polyunsaturated fatty acid-rich vegetable oils (21). Elaidic acid (EA, 9t18:1,Trans-9-Octadecenoic acid) is the main isomer of TFA, accounting for 80–90% of the total TFA content (22). The addition of EA can make food get better taste and longer shelf-life, so it was often used as a food additive. However, since the 1950s, there has been increasing evidence that EA intake is associated with cardiovascular disease and type 2 diabetes (23–25). Both a observational study (26) and a short-term randomized trial (27) have shown that systemic inflammation may be a potential mechanism between EA consumption and diabetes and coronary artery disease. Some countries have promulgated restrictions or completely prohibited food companies from adding EA to foods. However, not every country pays attention to this problem, and EA may be ingested by us unconsciously. Meanwhile, recent studies have found that EA consumption in the diet has a negative impact on the oral condition of mice, associated with periodontal bleeding (28). And for patients after periodontal treatment, banning EA-rich food intake can improve the periodontal prognosis (29).

No previous cross-sectional studies have investigated the relationship between plasma EA and moderate/severe periodontitis. Our aim was to examine the relationship between plasma EA and moderate/severe periodontitis using data from the National Health and Nutrition Examination Survey (NHANES). The NHANES is a national initiative that uses interviews, physicals, and oral exams to collect health data from representative samples of the American population [CDC, National Center for Health Statistics, (30)]. It measures the concentration of EA in plasma from thousands of individuals. Additionally, it includes standardized periodontal examinations performed by dentists, which improves the precision of periodontitis diagnosis. As a result, we carried out the research using the only NHANES plasma EA examination cycle in almost two decades (2009–2010), and we controlled for confounding variables like sociodemographic variables, lifestyle variables, and systemic disease. And in view of the relationship of the role of EA in promoting inflammation, it is assumed that the level of EA in plasma of patients with moderate/severe periodontitis may be higher. In our research, understanding the relationship between periodontitis and plasma EA may help dentists and policymakers create periodontitis prevention strategies that are effective.

## Materials and methods

This is a cross-sectional study that uses the published NHANES data set. The NHANES is a continuous national representative sample survey of the US population. Every 2 years since 1999, NHANES data have been made available. Every year, the poll evaluates a sample of 5,000 persons who are roughly nationally representative. These individuals are dispersed around the nation’s counties and travel to 15 of them annually. We used a survey cycle of NHANES 2009–2010. Details are available elsewhere (30). The agreement required all participants to provide informed consent, and the National Center for Health Statistics’ ethical review committee approved it. As these data are public, the research is exempt from additional approval from the ethical review committee of the local institution. Detailed information about NHANES laboratory/medical technologist methods, anthropometry, laboratory procedures, blood sample collection, stockpiling, diagnostics and quality standards are described elsewhere (31–34) (accessed 19 August 2013).^1^

The inclusion criteria included participants who received full periodontal examination (FMPE) and had plasma EA measurements. According to the NHANES guidelines, only subjects 30 years of age and older received a periodontal examination (35). Exclusion criteria include: (1) Incomplete plasma EA test or outliers of plasma EA (Based on variable distribution lookup: find outliers in data by looking at maximum and minimum values); (2) Individuals with the age is less than 30 years old, missing teeth and no periodontal measurement data. A total of 10,537 people were screened, of which 1,610 were interviewed by sampling (Supplementary Figure 1).

### Periodontal examination and classification

In the discovery dataset (NHANES 2009–2010), each participant’s full mouth (four quadrants) was evaluated for no/mild or moderate/severe periodontitis. All participants were examined by trained and calibrated dentists. The probing pocket depth (PPD) and clinical attachment loss (CAL) both show good reliability; interclass correlation coefficients (ICCs) range from 0.80 to 0.90 and 0.79 to 0.86, respectively. Periodontists probed six sites per tooth (mesio-, mid-, disto-buccal and mesio-, mid-, disto-lingual) during the periodontal examination. In order to assess periodontal health, only 28 teeth and 168 sites per individual could be examined due to the exclusion of third molars.

For the classification of periodontal disease, we used the CDC/AAP (Centers for Disease Control and Prevention and American Academy of Periodontology) case definitions (36). The term “no or mild periodontitis” was defined as no evidence of moderate, or severe periodontitis; Moderate/Severe: ≥ 2 interproximal areas, CAL ≥ 4 mm (not on the same tooth), or ≥ 2 interproximal areas, PPD ≥ 5 mm; the participants were divided into two groups based on their level of periodontitis, no/mild and moderate/severe (34).

### Plasma elaidic acid

Blood samples were taken from participants’ anterior elbow veins by professional doctors to evaluate the concentration of selected TFA in plasma. This is part of the NHANES agreement. Following acidic and basic hydrolysis, fatty acids in plasma are converted to free fatty acids. Liquid-liquid extraction was used to extract the free fatty acids, which were then derivatized with pentafluorobenzyl bromide (PFB-Br). Capillary gas chromatography was used to separate the fatty acids, and negative chemical ionization mass spectrometry was used to detect them. The specific mass to charge ratio of the ions formed in the ion source, as well as the chromatographic retention times of fatty acids, are then used to identify them. After that, the retention times are compared to those obtained using previously defined criteria. Internal standards were stable isotope-labeled fatty acids, which were quantified using standard solutions. This measurement procedure was used to determine 29 fatty acids and calculate TFA as a percentage of total fatty acids. These fatty acids constitute 95 percent of all fatty acids found in plasma. The approach describes four TFAs, one of which is EA (37).

### Potential confounders

Sociodemographic variables were age, gender, body mass index (BMI) (38), race/ethnicity, education level, and annual household income. We stratified the age of the continuous variables into ≤ 60 years, > 60 years and Separate non-Hispanic whites and others by race/ethnicity (39). Then, we stratified how many days use dental floss/device: 0–1, 2–4, and ≥ 5 days in the past week (40). Education levels were lower than high school, bachelor’s degree, and above, and family income was divided into three categories: under $20,000, between $20,000 and $75,000, and over $75,000. Lifestyle variables included smoker (smoked at least 100 cigarettes in life), drinker (have had at least 12 alcoholic drinks in 1 year), how healthy is the diet and vitamin C intake (on the first day’s total nutrient intake) (41). Systemic disease with a doctor diagnosis (hypertension, diabetes, sleep disorder, high cholesterol level, nephrasthenia). We opted to adapt our analysis to account for these potential confounding factors because periodontal disease may be linked to the covariates described above (10, 42–44).

### Statistical analysis

According to the standard of CDC guidelines, the data were statistically analyzed.^2^ The mean and standard deviation were used to describe continuous variables with a normal distribution, while categorical variables were stated in frequency or percentage. In order to know whether the plasma EA concentration of the participants included in the study is related to the severity of periodontitis, we divided the statistical analysis into three main steps. To begin, the plasma EA concentrations were separated into three groups based on the tertile level, and the baseline data distribution of participants in this study in various plasma EA concentrations (tertile and overall) was provided (45). The differences between the three tertile groups are demonstrated using the chi-square test (categorical variables), One-Way ANOVA (normal distribution), or Kruskal-Wallis test (skewed distribution). To calculate adjusted risk ratios and examine the relationship between plasma EA and moderate to severe periodontal disease, we used multivariable projected marginal proportions from logistic regression models in the second stage. There are three statistical models are constructed: model I, without adjusting covariates; Model II, only the age and gender are adjusted; Model III, adjust all covariates: Age; Gender; Race/ethnicity; BMI; Education level; Annual household income; Smoker; Drinker; Hypertension; Diabetes; Sleep disorder; High cholesterol level; Nephrasthenia; How many days use dental floss/device; How healthy is the diet, Vitamin C. Finally, we looked at age, gender, and race/ethnicity subgroups to see if there were any sources of heterogeneity.

It is worth noting that due to the missing data in NHANES database, we need to be filled for data integrity. If only complete cases are used for data analysis, the sample size will be insufficient, and our findings may be biased. Therefore, we use mean interpolation to the missing values (co-variates of continuous variables), whose main purpose is to maximize the statistical ability and avoid the loss of samples as much as possible. We conducted a sensitivity analysis to ensure the accuracy of the data analysis. All analyses are made by statistical software R (R Foundation)^3^ and Empower Stats.^4^ *P*-value less than 0.05 (two sides) is regarded as statistically significant.

## Results

### Characteristics of the study sample

Baseline characteristics for study participants were divided into three groups (Tertiles, T1-T3) by plasma EA level (Table 1). Gender, diabetes, sleep disorder and nephrasthenia were similar in the different groups of plasma EA (all *p*-values > 0.05).

**TABLE 1:** Characteristics of participants (*N* = 1,610).

Characteristic	Plasma EA, μmol/L				*P*-value
	Overall	T1 (2.66–10.60)	T2 (10.70–16.30)	T3 (16.40–39.40)	
No. of participants	1,610	534	534	542	
Plasma EA (μmol/L, mean ± SD)	14.80 ± 7.03	8.06 ± 1.70	13.41 ± 1.60	22.82 ± 5.53	< 0.001
Age (years, mean ± SD)	52.11 ± 14.21	50.39 ± 13.79	51.82 ± 14.13	54.08 ± 14.47	< 0.001
Vitamin C (mg, mean ± SD)	85.15 ± 92.43	95.70 ± 101.91	81.71 ± 83.41	78.16 ± 90.28	0.004
Age group, *n* (%)					0.020
≤60 years	1,138 (70.68%)	397 (74.34%)	380 (71.16%)	361 (66.61%)	
>60 years	472 (29.32%)	137 (25.66%)	154 (28.84%)	181 (33.39%)	
Gender, *n* (%)					0.110
Male	770 (47.83%)	275 (51.50%)	243 (45.51%)	252 (46.49%)	
Female	840 (52.17%)	259 (48.50%)	291 (54.49%)	290 (53.51%)	
BMI (Kg/m^2^,mean ± SD)	29.50 ± 6.69	27.53 ± 5.65	29.68 ± 6.87	31.25 ± 6.94	< 0.001
Race/ethnicity, *n* (%)					0.012
Non-Hispanic whites	752 (46.71%)	227 (42.51%)	246 (46.07%)	279 (51.48%)	
Others	858 (53.29%)	307 (57.49%)	288 (53.93%)	263 (48.52%)	
Education level (%)					< 0.001
≤ High school	783 (48.63%)	207 (38.76%)	233 (43.63%)	343 (63.28%)	
College	447 (27.76%)	147 (27.53%)	164 (30.71%)	136 (25.09%)	
> College	376 (23.35%)	180 (33.71%)	135 (25.28%)	61 (11.25%)	
Missing data	4 (0.25%)	0 (0.00%)	2 (0.37%)	2 (0.37%)	
Annual household income, *n* (%)					< 0.001
<20,000$	273 (16.96%)	65 (12.17%)	91 (17.04%)	117 (21.59%)	
20,000–75,000$	760 (47.20%)	225 (42.13%)	258 (48.31%)	277 (51.11%)	
>75,000$	66 (4.10%)	25 (4.68%)	26 (4.87%)	15 (2.77%)	
Missing data	511 (31.74%)	219 (41.01%)	159 (29.78%)	133 (24.54%)	
Smoker, *n* (%)					0.009
Yes	673 (41.80%)	206 (38.58%)	212 (39.70%)	255 (47.05%)	
No	937 (58.20%)	328 (61.42%)	322 (60.30%)	287 (52.95%)	
Drinker, *n* (%)					0.038
Yes	1,089 (67.64%)	362 (67.79%)	365 (68.35%)	362 (66.79%)	
No	405 (25.16%)	126 (23.60%)	125 (23.41%)	154 (28.41%)	
Missing data	116 (7.20%)	46 (8.61%)	44 (8.24%)	26 (4.80%)	
Hypertension, *n* (%)					0.003
Yes	582 (36.15%)	176 (32.96%)	175 (32.77%)	231 (42.62%)	
No	1,026 (63.73%)	358 (67.04%)	358 (67.04%)	310 (57.20%)	
Missing data	2 (0.12%)	0 (0.00%)	1 (0.19%)	1 (0.18%)	
Diabetes, *n* (%)					0.142
Yes	185 (11.49%)	62 (11.61%)	46 (8.61%)	77 (14.21%)	
No	1,386 (86.09%)	460 (86.14%)	474 (88.76%)	452 (83.39%)	
Borderline	37 (2.30%)	11 (2.06%)	14 (2.62%)	12 (2.21%)	
Missing data	2 (0.12%)	1 (0.19%)	0 (0.00%)	1 (0.18%)	
Sleep disorder, *n* (%)					0.600
Yes	119 (7.39%)	38 (7.12%)	44 (8.24%)	37 (6.83%)	
No	1,487 (92.36%)	494 (92.51%)	488 (91.39%)	505 (93.17%)	
Missing data	4 (0.25%)	2 (0.37%)	2 (0.37%)	0 (0.00%)	
High cholesterol level, *n* (%)					< 0.001
Yes	536 (33.29%)	141 (26.40%)	185 (34.64%)	210 (38.75%)	
No	704 (43.73%)	287 (53.75%)	229 (42.88%)	188 (34.69%)	
Missing data	370 (22.98%)	106 (19.85%)	120 (22.47%)	144 (26.57%)	
Nephrasthenia, *n* (%)					0.639
Yes	30 (1.86%)	8 (1.50%)	8 (1.50%)	14 (2.58%)	
No	1,573 (97.70%)	524 (98.13%)	523 (97.94%)	526 (97.05%)	
Missing data	7 (0.43%)	2 (0.37%)	3 (0.56%)	2 (0.37%)	
How many days use dental floss/device					< 0.001
1	627(38.94%)	180 (33.71%)	213 (39.89%)	234 (43.17%)	
2–4	420(26.09%)	132 (24.72%)	136 (25.47%)	152 (28.04%)	
5–7	563(34.97%)	222 (41.57%)	185 (34.64%)	156 (28.78%)	
How healthy is the diet					< 0.001
Excellent	128(7.95%)	70 (13.11%)	35 (6.55%)	23 (4.24%)	
Very good	319(19.81%)	141 (26.40%)	106 (19.85%)	72 (13.28%)	
Good	714(44.35%)	211 (39.51%)	237 (44.38%)	266 (49.08%)	
Fair	381(23.66%)	101 (18.91%)	127 (23.78%)	153 (28.23%)	
Poor	67(4.16%)	10 (1.87%)	29 (5.43%)	28 (5.17%)	
Don’t know	1(0.06%)	1 (0.19%)	0 (0.00%)	0 (0.00%)	
CDC/AAP case definition, *n* (%)					< 0.001
Non/mild periodontitis	876 (54.41%)	315 (58.99%)	309 (57.87%)	252 (46.49%)	
Moderate/severe periodontitis	734 (45.59%)	219 (41.01%)	225 (42.13%)	290 (53.51%)	

AAP, American Academy of Periodontology; CDC, Centers for Disease Control and Prevention; T, Tertile; BMI, Body mass index; EA, elaidic acid.

The average age of the participants was 52.11 years, 770 (47.83%) were male and 840 (52.17%) were female. A total of 45.59% of participants have moderate or severe periodontitis. Compared with T1 and T2, lower proportion of T3 participants ≤ 60 years of age. Participants in T3 (individuals with high plasma EA concentrations) were more likely to be non-Hispanic white, less educated, to floss/use devices on a relatively infrequent basis, to need to improve their dietary health, to consume less vitamin C, and to have lower annual household income as compared to T1 (individuals with low plasma EA concentrations). In addition, they smoke and drink more, and have higher BMI, hypertension prevalence rate, cholesterol levels and moderate/severe periodontal disease prevalence rate.

### Plasma elaidic acid and periodontal disease

Table 2 lists the association between plasma EA level and moderate/severe periodontitis. The model I showed that plasma EA level increased the odds of moderate/severe periodontitis, with OR 1.029 (CI: 1.014, 1.043), *P* = 0.00009. A similar result was detected after adjusting the age group and gender in model II. Under the completely adjusted model (model III), adjusting age group, gender, race/ethnicity, BMI, education level, annual household income, smoker, drinker, hypertension, diabetes, sleep disorder, high cholesterol level, nephrasthenia, how many days use dental floss/device; how healthy is the diet, and vitamin C intake. The model III showed that plasma EA level increased the odds of moderate/severe periodontitis, with OR = 1.021 (95%CI: 1.004–1.039), *P* = 0.01394. It is means that for every unit of plasma EA level increased, the odds of moderate/severe periodontitis increased by 2.1%.

**TABLE 2:** Multivariate logistic regression model for association between plasma EA (umol/L) and moderate/severe periodontitis in different models.

Exposure	Model I (OR, 95% CI, *P*-value)		Model II (OR, 95% CI, *P*-value)		Model III (OR, 95% CI, *P*-value)	
Plasma EA (umol/L)	1.029 (1.014, 1.043)	0.00009	1.028 (1.013, 1.043)	0.00021	1.021 (1.004, 1.039)	0.01394
**Plasma EA (umol/L)**						
T1 (2.66–10.60)	1.0		1.0		1.0	
T2 (10.70–16.30)	1.047 (0.821, 1.336)	0.70951	1.068 (0.827, 1.379)	0.61555	1.075 (0.814, 1.420)	0.60835
T3 (16.40–39.40)	1.655 (1.300, 2.107)	0.00004	1.660 (1.288, 2.139)	0.00009	1.519 (1.136, 2.030)	0.00477
*p*-value for trend	0.00004	0.00008			0.00452	

CI, confidence interval.

Model I adjust for: None. Model II adjust for: Age; Gender.

Model III adjust for: Age; Gender; Race/ethnicity; Body mass index; Education level; Annual household income; Smoker; Drinker; Hypertension; Diabetes; Sleep disorder; High cholesterol level; Nephrasthenia; How many days use dental floss/device; How healthy is the diet, Vitamin C.

To ensure the results’ robustness, convert continuous independent variables into classified variables. The plasma EA levels were stratified into categorical variables according to the Tertile, and the trend P was estimated (Table 2). In the full adjustment model, the estimated risk rate of plasma EA levels in T2 and T3 were 7.5 and 51.9%, respectively, as compared to the reference T1, and the trend *P* = 0.00452.

### Subgroup analyses

**FIGURE 1:**
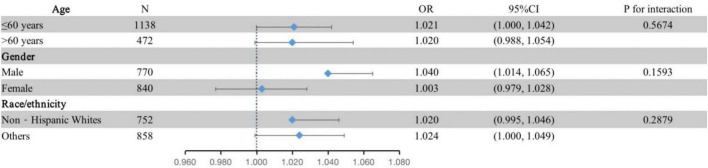
Subgroup analyses of the associations between the tertiles of plasma EA and moderate/severe periodontitis by age, gender and race/ethnicity in the discovery dataset (NHANES 2009–2010; *n* = 1,610). The adjustment model was the same as model III in Table 2 except that the covariates tested as effect modifiers were not adjusted. Periodontitis is defined by the CDC/AAP case definition.

The subgroup analysis was conducted to identify possible sources of heterogeneity. The interaction term did not reach significance (P _interaction_ > 0.05) when analyses were stratified by age, gender and race/ethnicity (Figure 1). Interaction analysis showed a stronger association between plasma EA levels and moderate/severe periodontitis in the male population (OR = 1.040, 95% CI: 1.014–1.065), although there was no statistical significance for the interaction (P _interaction_ = 0.1593).

## Discussion

According to this population-based observational study (NHANES 2009–2010), plasma EA is associated to moderate/severe periodontitis in American adults. The primary findings remained consistent and robust across all validations and sensitivity analyses after adjustment for a variety of potential confounders. It’s interesting that we discovered higher odds of periodontitis in participants with higher tertile. This means that target groups with high EA consumption, such as those with food insecurity and obesity, should prioritize EA restrictions (46, 47). In addition, compared with the participants in the lowest tertile, the percentage of participants in the highest tertile who are younger than 60 years old is lower. This could be because the use of TFAs (EA) in the food sector has long been unrestricted, resulting in higher cumulative EA intake among the elderly (48).

The association between TFA and a pro-inflammatory dietary pattern as well as the relationship between a pro-inflammatory dietary pattern and periodontitis are increasingly known (19, 39). This relationship is the premise of our study hypothesis that TFA as a typical pro-inflammatory food may be potential odds for periodontitis. A cross-sectional study found that people who consumed more TFA had an increased percentage of bleeding sites on periodontal probing (49). And in a prospective study, the diet of patients undergoing non-surgical periodontal treatment was optimized for 4 weeks (intake of vitamins, fiber, and restriction of TFA intake). This study found that the reduction of full-mouth bleeding score and PPD was more significant in the population with optimized diet than in the group with only periodontal treatment (29). In a cross-sectional study of patients with stable coronary artery disease (50), it was found that dietary patterns of patients with periodontitis favor the eating of fried foods, which are known to be rich in TFA. However, these clinical studies contain too many confounding factors, and whether TFA play a real role in it is not known.

Recently reported studies have found that EA has pro-inflammatory potential, which seems to be able to link this result with TFAs, pro-inflammatory dietary pattern and the relationship with periodontitis (21). The pro-inflammatory effect may be the reason why it becomes odds factor for periodontitis. EA has been demonstrated in animal models to increase c-reactive protein (CRP) in blood and cerebrospinal fluid (51). It is incorporated into endothelial cells (EC) in a dose-dependent manner, and most likely plays a major role in inflammation after incorporation into cell membranes (52). It stimulates the development of pro-inflammatory biomarkers including increased intercellular cell adhesion molecule I (ICAM-1) and nuclear factor- κB (NF- κB) (53). The activation of toll like receptors 4 (TLR4) in the cell membrane is thought to be responsible for the production of inflammation in EC by EA (54). It then causes mitogen-activated protein kinases (MAPKs) to be phosphorylated, which increases the release of inflammatory proteins like Interleukin-6 (IL-6), Vascular cell adhesion molecule-1, and ICAM-1 mRNA. Another mechanism of EA is that it can deeply activate the NF-κB signal, manifested as an approximately twofold increase in inhibitor of NF-κB phosphorylation, followed by an increase in IL-6 concentration and TNFα expression (55). Notably, circulating inflammatory mediators such as TNF-α, IL-6 and CPR serve as the link between systemic chronic inflammatory diseases and chronic periodontitis, and better early diagnosis of periodontitis by these inflammatory biomarkers (56, 57). In conclusion, EA may increase the odds of periodontitis if it significantly affects systemic inflammation and leads to chronic inflammatory diseases.

The approach we looked at has some drawbacks. We are unable to more thoroughly assess the impact of plasma EA on periodontitis because of the limited amount of data available in the NHANES database. Second, because our results are based on the population of the United States, they might not be applicable to other nations. Finally, a cross-sectional study is the type of research design used. We are unable to determine a causal link between plasma EA levels and moderate or severe periodontitis due to the method’s inherent limitations.

At the same time, our study has some advantages. Firstly, the periodontal diagnosis is based on a comprehensive examination of six parts of each tooth, which is considered the gold standard method (58). Secondly, because NHANES data was collected on all 7 days of the week throughout the year, the possibility of selection bias is quite minimal. And unlike data of dietary retrospective interview, data for plasma EA levels avoid the potential for recall bias.

## Conclusion

Our study found that moderate/severe periodontitis in adults in the United States may be associated with plasma EA levels. For target groups with high EA consumption such as food insecurity and obesity, the periodontal health benefits of reducing EA intake may be more pronounced. Those findings provide further support for the recommendation to limit trans-fatty acid consumption. The relationship between EA levels in plasma and periodontitis needs more studies to confirm.

## Data availability statement

Publicly available datasets were analyzed in this study. This data can be found here: https://wwwn.cdc.gov/nchs/nhanes/.

## Author contributions

HJ designed the research, contributed to data interpretation, and drafted the manuscript. CZ, YJ, JF, PQ, and SQ contributed to data interpretation and the manuscript. LY and SJ reviewed the article. All authors read and approved the final manuscript.

## Footnotes

1
http://www.cdc.gov/NCHS/data/nhanes/nhanes_09_10/CRP_F_met.pdf

2
https://wwwn.cdc.gov/nchs/nhanes/tutorials/default.aspx

3
http://www.rproject.org

4
http://www.empowerstats.net/cn/download.html
