# Supplementary figures and images for: Plasma elaidic acid level is associated with periodontal health in American adults: A cross-sectional study
Source: Front Nutr. 2022 Dec 8;9:1034841. doi: 10.3389/fnut.2022.1034841 (PMC9773206; doi:10.3389/fnut.2022.1034841)

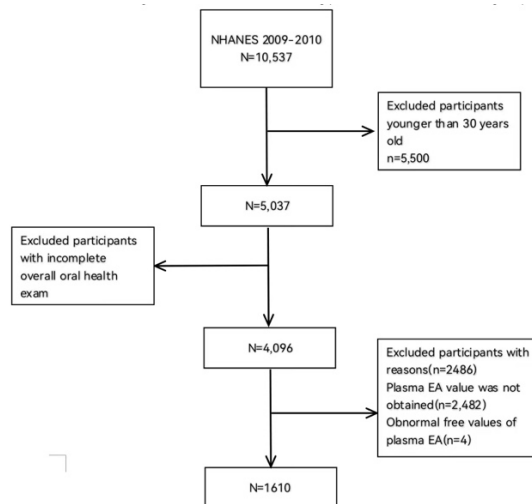

Figure S1. Characteristics of participants in the validation dataset (NHANES 2009–2010)

Supplement: Supplementary file 1 [file Data_Sheet_1.PDF]
